# Role of Hyaluronic Acid in Selected Malignant Neoplasms in Women

**DOI:** 10.3390/biomedicines11020304

**Published:** 2023-01-21

**Authors:** Anna Markowska, Michał Antoszczak, Janina Markowska, Adam Huczyński

**Affiliations:** 1 Department of Perinatology and Women’s Health, Poznań University of Medical Sciences, 60-535 Poznań, Poland; 2 Department of Medical Chemistry, Faculty of Chemistry, Adam Mickiewicz University, 61-614 Poznań, Poland; 3 Department of Oncology, Gynecological Oncology, Poznań University of Medical Sciences, 60-569 Poznań, Poland

**Keywords:** hyaluronan, breast cancer, cervical cancer, endometrial cancer, ovarian cancer

## Abstract

Hyaluronic acid (HA) is a significant glycosaminoglycan component of the extracellular matrix, playing an essential role in cell localization and proliferation. However, high levels of HA may also correlate with multidrug resistance of tumor cells, an increased tendency to metastasize, or cancer progression, and thus represent a very unfavorable prognosis for cancer patients. The purpose of this review article is to summarize the results of studies describing the relationship between HA, the main ligand of the CD44 receptor, or other components of the HA signaling pathway. In addition, we review the course of selected female malignancies, i.e., breast, cervical, endometrial, and ovarian cancer, with the main focus on the mechanisms oriented to CD44. We also analyze reports on the beneficial use of HA-containing preparations in adjuvant therapy among patients with these types of cancer. Data from the literature suggest that HA and its family members may be critical prognostic biomarkers of selected malignancies among women. Nevertheless, the results of the available studies are inconclusive, and the actual clinical significance of HA expression analysis is still quite enigmatic. In our opinion, the HA-CD44 signaling pathway should be an attractive target for future research related to targeted therapy in gynecological cancers.

## 1. Introduction

**Figure 1:**
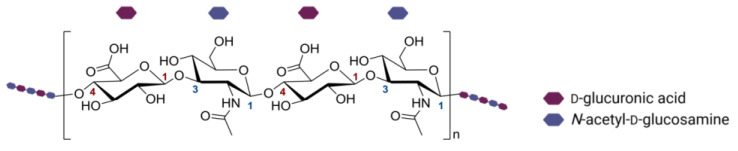
Structure of hyaluronic acid. The figure was created with BioRender.com, accessed on 28 November 2022.

**Figure 2:**
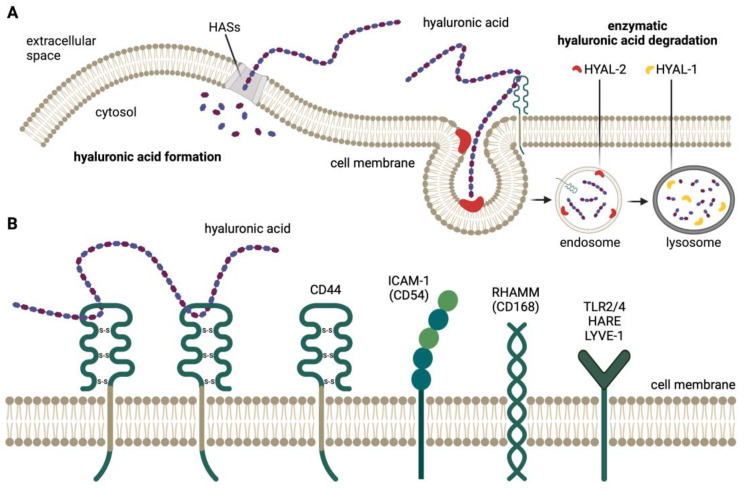
(**A**) Formation and enzymatic degradation of hyaluronic acid by hyaluronate synthases and hyaluronidases, respectively; and (**B**) interaction between hyaluronic acid and its receptors. Abbreviations used: CD44, cluster of differentiation 44; HARE, hyaluronan receptor for endocytosis; HASs, hyaluronate synthases; HYAL-1/2, hyaluronidase 1 or 2; ICAM-1, intercellular adhesion molecule 1; LYVE-1, lymphatic vessel endothelial hyaluronan receptor 1; RHAMM, receptor for hyaluronan-mediated motility; TLR2/4, Toll-like receptors 2 and 4. The figure was created with BioRender.com, accessed on 28 November 2022.

Hyaluronic acid (HA) is a polysaccharide of a linear structure belonging to the glycosaminoglycan group, consisting of repeating disaccharide units—D-glucuronic acid and *N*-acetyl-D-glucosamine—linked by β(1→4) and β(1→3) glycosidic bonds (Figure 1) [1]. HA is produced on the inner surface of the cell membrane with the participation of hyaluronate synthases—membrane-bound enzymes (Figure 2A). The expression of these enzymes is influenced by various growth factors (e.g., EGF, bFGF) and selected cytokines (e.g., IL-1, TNFα) [2,3]. The degradation of HA also occurs through the enzymatic action of hyaluronidases (endoglycosidases) [1,4]; such specified enzymes are localized in different locations (HYAL-2 in inner part of endosome membranes, HYAL-1 in lysosomes) and initiate the degradation of HA chains (Figure 2A). This polysaccharide is present in the human body, including such structures as the umbilical cord, connective tissue, synovium, intervertebral discs, and vitreous body [2]. It has hygroscopic properties; by binding water in the dermis (HA can bind up to 250 water molecules), it gives the skin the right elasticity and flexibility.

The extracellular matrix is a complex network of macromolecules that provides a critical mechanical scaffold for its components. It also mediates many vital biochemical processes. One of the major components of the extracellular matrix is HA. By modulating intercellular interactions, it is involved in immune processes, angiogenesis, regulation of anti-inflammatory factors, and signaling. The biological activity of HA depends on attachment to hyaluronan binding proteins (HABPs) (Figure 2B). This group includes the proteins bound to the cell membrane (e.g., CD44, TLR2/4, HARE), as well as the proteins that are part of the extracellular matrix [5,6,7]. The main HA receptor is CD44 protein (cluster of differentiation 44), a single-chain trans-membrane glycoprotein (Figure 2B), which is also one of the most relevant markers of cancer stem cells (CSCs) in many types of cancer [8,9,10]. The CD44 receptor (in all its isoforms) has been shown to have a domain that binds with exceptionally high affinity to HA as a ligand. This property can be exploited to easily and efficiently isolate cells with cancer stem cell characteristics, among other actions, thereby promoting new anticancer treatment strategies targeting CSCs [11,12]. CD44 can also interact with collagen, laminin, or fibronectin, which are involved in cell adhesion. This affects the motility and migration of tumor cells, which are essential in cancer metastasis [9,13,14,15]. Besides CD44, HA can also bind to other classes of cell-surface receptors, including ICAM-1 (aka CD54) (intercellular adhesion molecule 1), RHAMM (aka CD168) (receptor for hyaluronan-mediated motility), and others (Figure 2B), modulating a range of intracellular signals [16,17,18]. 

Although HA regulates many normal physiological processes, this polysaccharide can also contribute to the development of chronic and acute diseases, including cancer. In the course of cancer development, HA affects, among other things, the migration, invasiveness, and resistance of cancer cells to the chemotherapeutic drugs applied [4,5]. HA is a factor involved in carcinogenesis [5,6,19] through its interaction with specific receptors and intracellular signal transduction. HA regulates the microenvironment of tumors, thus promoting their malignant phenotype. High levels of HA have been identified in breast, lung, ovarian, and prostate cancer, among others [20,21,22]. It has been experimentally demonstrated that other components of the HA signaling pathway, including hyaluronate synthases (HASs), hyaluronidase HYAL-1, and protein receptors, can also promote malignant behavior of tumor cells in vitro, in addition to tumor growth, metastatic capacity, and angiogenesis in animal models [23,24,25,26,27,28,29,30]. Specifically, HYAL-1 is able to degrade HA into fragments with proangiogenic and multidrug resistance-inducing properties [31], which suggests the potential utility of this enzyme as an independent predictor of metastasis [32,33,34,35]. For this reason, HA and its family members have attracted the attention of the scientific community as extremely interesting diagnostic and/or prognostic markers in the course of many types of cancer [33,34,36,37,38,39,40,41,42,43,44].

Since the discovery of CD44 receptor overexpression in many types of solid tumors, a very interesting direction of research is to develop innovative HA-based drug delivery systems, exhibiting preferential accumulation in tumors and increased cell uptake. Until now, many authors have been concerned with HA’s tumor-targeting ability [45]. For example, HA-based delivery systems have been used to improve the selectivity of standard cytotoxic drugs against ovarian cancer cells [46,47], including an HA-paclitaxel hybrid with increased in vivo efficiency on ovarian cancer cells after intraperitoneal injection, compared to that of free anticancer drugs [48]. 

Despite a growing number of reports, to the best of our knowledge, there are no articles summarizing the results of studies on the relationship between HA levels (serum, plasma, or stromal HA levels) and the risk of developing or progressing selected malignancies in women. Therefore, in order to verify the potential clinical significance of HA and other components of the signaling pathway of this polysaccharide, in this review article, we focus on establishing its potential role as a biomarker in breast, cervical, endometrial, and ovarian cancer. We specifically discuss the interaction of hyaluronan and CD44 ligands in these female malignancies. Additionally, we were also interested in reports on the potential therapeutic effects of using HA-containing preparations in a group of patients with gynecologic cancers. To this end, we ran an in-depth search of the Google Scholar and PubMed databases for original papers describing the relationship between HA levels, the expression of HA family members, and the course of female malignancies. These papers were published mainly in the last two decades (Table 1). The keywords used were hyaluronic acid, hyaluronan, breast cancer, cervical cancer, endometrial cancer, and ovarian cancer. Additional published papers were obtained by checking the references of the screened articles.

## 2. Breast Cancer

Breast cancer (BC) is the most common malignant tumor in women worldwide. In 2020, BC was diagnosed in nearly 2.5 million patients, accounting for nearly ¼ of all malignancies diagnosed in women [70,71]. Conventional tumor markers are still of limited use, not only in planning oncological treatment, but also in monitoring its efficacy for patients with metastatic BC. For this reason, the search for new tumor markers is an extremely important task to be solved by modern oncological medicine, and in this context, raised HA levels are increasingly frequently being linked to progression and unfavorable course of BC. Recent findings further indicate that the disruption of metabolic reprogramming induced by the epithelial–mesenchymal transition (EMT) may affect HA production and, thus, reorganize the extracellular matrix, which, in turn, may contribute to inhibiting the progression of aggressive forms of BC [72].

In a study conducted by Delpech et al. [49], HA levels were measured in a group of 83 patients diagnosed with BC (57 women with systemic metastasis and 26 women without metastasis), and the results were compared with those obtained for 50 patients with non-cancerous breast disease. Interestingly, HA levels were higher in the serum samples from women with metastatic cancer compared to those in the patients without detected metastases (*p* < 0.0001), while the lowest HA levels were identified in the samples from the control group (*p* < 0.01) [49]. At the same time, the number of metastases was irrelevant to HA levels [49]. Three months after the start of oncological treatment, reduced values of HA levels were noted in the group of women with metastases who responded positively to the applied chemotherapy; however, the initial serum HA level was not directly indicative of the effectiveness of the applied treatment [49]. Another study found not only elevated HA levels in the serum of BC patients before treatment, but also increased activity of the enzymes that synthesize this polysaccharide compared to the control group; after the first cycle of chemotherapy, these indicators were significantly reduced (*p* < 0.001) [51]. On the other hand, Peng et al. [50] determined plasma HA levels in patients with metastatic BC in relation to progression-free survival (PFS) and overall survival (OS). According to the authors of that study, a high plasma HA level was clearly associated with a poor prognosis for cancer patients, while a decrease in HA levels correlated with a good response to the applied treatment, as assessed radiologically after the first cycle of chemotherapy (AUC 0.79) [50].

A meta-analysis including 2664 BC cases indicated that high HA levels correlated with shortened OS (HR 1.86, 95% CI 1.28–2.71, *p* = 0.001), as well as disease-free survival (DFS), recurrence-free survival (RFS), and PFS (HR 1.63, 95% CI 1.14–2.33, *p* = 0.007) [73]. Moreover, high plasma (HR 3.26, 95% CI 2.25–4.73, *p* < 0.001) and stroma (HR 1.63, 95% CI 1.06–2.51, *p* = 0.025) HA levels were correlated with shorter OS and associated with the presence of lymph node metastasis (HR 1.55, 95% CI 0.96–2.49, *p* = 0.070) or more advanced disease (HR 2.10, 95% CI 0.89–4.96, *p* = 0.089) [73]. The correlation between the survival time of BC patients with HABP1 overexpression and clinicopathological factors has also been studied [52]. It was observed that the survival rate of patients with low expression of HABP1 was significantly higher than that of the patients with high expression of this protein (*p* = 0.025), while the level of HABP1 expression in tumor cells was higher than in normal cells [52]. Jiang et al. [74] investigated the association of single nucleotide polymorphisms (SNPs) with HABP1 and BC characteristics in female residents of northern China. On the basis of their results, SNPs of the minor allele rs2285747 of HABP1 was found to be associated with an increased risk of BC and elevated expression of this protein in the study population [74].

In MCF-7 drug-resistant BC cells, an increase in HA production was observed due to the upregulation of HA synthase-2 (HAS2), while the upregulation of HAS2 contributed to the chemoresistance of cancer cells, as well as their ability to form drug-resistant spheres, through activation of the CD44/Nrf2 signaling pathway [75]. HAS2 levels were also correlated with the malignant phenotype of BC cells [53]. In contrast, a study by Gao et al. [76] has shown that the bifunctional enzyme PFKFB4, whose expression is elevated in many types of cancer, promotes the metastatic capacity of BC cells as a result of inducing HAS2 expression and HA production in a p38-dependent manner. Compared to non-cancerous HBL-100 cell lines and normal breast tissues, elevated levels of HYAL-1 were detected in the BC cell lines MCF-7 and MDA-MB-231 [54]. Intriguingly, lowering the activity of this enzyme not only resulted in reduced growth, adhesion, and potential for invasion and angiogenesis of tumor cells in vitro, but also inhibited tumor growth and microvessel density in animal models [54].

On the other hand, the results of a randomized phase III trial indicated that there was no apparent difference in the treatment of acute dermatitis among BC patients using HA during radiation therapy compared to the group using a simple emollient; however, the beneficial effects of HA included a contribution to a reduction in pain sensation (*p* = 0.053) and a 20% reduction in colorimetric levels (*p* = 0.46) [77]. Similarly, unfavorable results were obtained in another study, which found no benefit of topical HA-based gel in reducing the development of radiodermatitis among BC patients after complementary radiotherapy when compared to a group using Vaseline gel [78]. A detailed meta-analysis of randomized controlled trials involving 500 BC patients, on the other hand, showed some ambiguity in the results obtained in this regard [79]. HA was more effective in reducing the risk of radiodermatitis compared to phytosterol, omega-3/6/9 acids, and vitamin E. However, other studies indicated that the effectiveness of HA was comparable to that of grapevine extract and thermal water, or inferior compared to other topical agents [79].

## 3. Cervical Cancer

Cervical cancer (CC) is the fourth most commonly diagnosed malignancy in women, with varying incidences in different geographic zones [71], which is due to the relatively long time required for significant changes to appear in the normal cervical epithelium following persistent HPV infection [80,81]. HA, as a component of the extracellular matrix, has been found to play an active role in inflammation, including in viral infections [8].

The HA-CD44 pathway may play an important role in CC [55]. Importantly, studies conducted on the HeLa cell line showed that natural polyphenols such as karanjin, plumbagin, and pongapin, which affect the inhibition of cell proliferation and induce apoptosis, also have the ability to attenuate the expression of the HA-CD44 pathway [55]. The important role of HA in the course of cancer is indicated by a meta-analysis of 22 studies from electronic databases involving more than 2200 patients with CC [82]. In the studied group of women, a correlation was determined between the expression levels of seven important CSC markers, including the CD44 protein, and clinical parameters DFS and OS [82]. Specifically, it was shown that overexpression of CD44, a major cell surface receptor for hyaluronan, was significantly associated with worse OS (HR 1.14, 95% CI 1.07–1.22, *p* = 0.0001) [82], suggesting that this marker could be used as a prognostic indicator of adverse survival among CC patients. In a study conducted by Zhang et al. [56], aimed at determining the role of HABP1 and its association with clinical features among CC patients, the overexpression of this protein was found to correlate with advanced FIGO stage (*p* = 0. 001), worse histologic grade (*p* = 0.013), larger tumor size (*p* = 0.025), lymphatic vessel invasion (*p* = 0.024), deeper infiltration of the lining (*p* = 0.001), and greater lymph node metastasis (*p* = 0.023). In addition, HABP1 overexpression was an independent factor for DFS (HR 3.082, 95% CI 1.372–7.501, *p* = 0.007) [56].

Although HA is involved in the promotion of cancer progression by its interaction with surface receptors of cancer cells, the experimental therapy with this polysaccharide may interfere with tumor invasion and have an inhibitory effect on tumor growth in vivo. Specifically, the in vitro studies conducted on the cancer cell line HeLa, as well as in vivo tests on an animal model, have shown that administration of HA and selenium in combination with the chemotherapeutic agent doxorubicin effectively inhibits tumor cell proliferation and induces programmed cell death through the Bcl-2 signaling pathway [83]. It should, however, be emphasized that doxorubicin is not the first drug of choice for CC. Similarly, in experiments on mice, paclitaxel in combination with HA proved more effective in inhibiting metastasis of U14 cervical tumors compared to the classical monochemotherapy with taxol [84]. The positive effects of such co-treatment might result from different mechanisms, including an increase in host immunity, drug delivery efficiency, or the saturation of the specific surface receptor(s) by HA, which prevented tumor cells from attaching to the extracellular matrix and finally led to their death [84].

A randomized, two-arm clinical trial involving 180 women treated with follow-up radiation therapy after surgery evaluated the effect of vaginal globules containing low-molecular-weight HA on the incidence of side effects [85]. Significantly, in the treatment arm, nearly 90% of the patients had no side effects related to the radiotherapy administered, such as inflammation, vaginal dryness, or dyspareunia, while in the control arm, all symptoms were moderate to severe [85]. In a study by Riemma et al. [86], 153 women with histologically confirmed low-grade squamous intraepithelial lesion (LSIL) on the cervix underwent a three-arm clinical trial using oral *Echinacea* extracts, commonly known for their immunomodulatory and anti-inflammatory activity, in combination with HA in the form of vaginal globules. Interestingly, in the group using *Echinacea angustifolia* and HA, a significant therapeutic effect was achieved as measured by standard parameters, namely colposcopic, histological, and clinical examination [86]. As shown in a study conducted by Patino et al. [87] concerning patients with CC, the use of intravesical-HA instillations may have beneficial effects on the bladder mucosa and prevent the development of acute radiation-induced cystitis (RIC) during radiation therapy.

## 4. Endometrial Cancer

Endometrial cancer (EC) is the sixth most common malignancy in women, with a steadily increasing incidence, especially in economically developed countries [71]. Only two drugs (dostarlimab and pembrolizumab) have been approved by the Food and Drug Administration (FDA) for anticancer therapy for this type of cancer since 1971 [62]. For the patients with advanced or recurrent disease, the prognosis is very poor, with CSCs being responsible for the cancer progression and the phenomenon of drug resistance [88]. It was established that HA levels, as well as CD44 expression, are markedly increased in patients with EC [10,88,89]. In addition, the HA-CD44 pathway, involved in developing early endometrial lesions [90], can lead to cancer development.

In a study by Paiva et al. [42], HA, its synthases, and its degradative enzymes were identified in EC with varying degrees of histological malignancy (grades I–III). The level of HA increased with increasing tumor malignancy, with the trend being significant only for tumors with grade II malignancy (*p* < 0.05) [42]. Immunohistochemical analysis of 343 tissue samples from normal, atrophic, hypertrophic, and cancerous endometrium to measure hyaluronidase activity showed that reduced HYAL-1 expression was associated with a higher degree of EC aggressiveness [60]. HYAL-1 and HYAL-2 enzymes were shown to be coexpressed and, at the same time, significantly downregulated in endometrioid EC, which also correlated with increased HA accumulation [58]. Zhao et al. [59] evaluated the expression of HABP1 protein in tissues taken from 188 patients with EC, as well as in benign endometrial lesions (43 patients) and 41 samples of normal endometrium. There was a significant (*p* < 0.001) increase in the expression of this protein in cancer tissues compared to in benign lesions and normal endometrium [59]. High expression was simultaneously associated with increased disease stage, infiltration of lymphatic spaces, and metastasis, as well as shorter DFS and OS [59]. In contrast, a study by Jiang et al. [6] conducted on tissue specimens from 370 women with EC (analysis of the data from patients suffering from EC) detected increased expression of the HABP2 protein; however, decreased expression of this protein was associated with longer OS. An association of HABP2 with increased histological maturity (malignancy) of cancer and residual tumor after surgery has been reported [6]. These studies indicate the possible utility of changes in HABP1/2 protein expression as diagnostic markers or independent prognostic factors in EC. Analysis of serum samples from 59 patients with EC and 22 healthy postmenopausal women further indicated that elevated HAS1 expression correlates with the depth of myometrial invasion, the degree of histologic malignancy of the tumor, or the involvement of the lymphovascular space [57]. The same trend was observed for elevated HA levels [57]. On the contrary, HAS2/3 overexpression showed little association with these parameters [57]. A study by Nykopp et al. [58] showed that the increase in immunoreactivity for HASs in tumor cells did not correlate with changes in tumor mRNA levels for HASs, which may suggest that a decreased turnover of HAS proteins may contribute to HA accumulation.

In 2020, the expression of the RHAMM protein [62], one of the cell surface receptors whose levels are elevated in many types of cancer, was studied. The study used patient tissues obtained from endometrial biopsies or hysterectomy specimens from women diagnosed with EC [62]. A detailed analysis of 225 cases of EC, including serous and endometrioid types, as well as 8 cases of normal endometrium, showed that RHAMM expression was markedly increased in serous EC characterized by a high degree of malignancy relative to its expression in less aggressive endometrioid forms [62]. RHAMM protein expression was also positively correlated with the stage of the disease [62]. In contrast, another study revealed that RHAMM positivity rates were 100% in the group of patients with positive lymph nodes, while among the patients with negative lymph nodes, these rates were around 51% (*p* < 0.01) [63]. Similar results indicating an association between the increased RHAMM receptor expression and the stage and aggressiveness of EC have also been obtained by other authors [61].

On the other hand, HA-containing formulations can help to improve the quality of life of women with a history of oncological treatment by reducing the bothersome side effects. The effect of topical HA therapy in women undergoing surgical treatment and subsequent vaginal brachytherapy has been described by assessment of the risk of inflammation or late symptoms (fibrosis, telangiectasia) whose appearance has been prevented by the use of HA [91]. In contrast, Murakami et al. [92] described the effect of locally applied HA-containing gel on the incidence of late rectal bleeding among gynecologic cancer patients undergoing image-guided adaptive brachytherapy (IGABT). Among 19 women with EC and 46 patients with CC, there was a statistically significant lower incidence of rectal bleeding compared to that in the women not receiving HA therapy (*p* = 0.01) [92]. On the other hand, a single-arm clinical trial conducted on a group of 43 patients who had undergone surgery for EC and subsequent radiation therapy showed the effectiveness of treatment with HA-based vaginal gel, including a reduction in symptoms of vaginal and vulvar discomfort and pain [93]. Carter et al. [94] showed that the use of a non-hormonal vaginal gel containing HA could help to alleviate vulvovaginal estrogen-deprivation symptoms, thereby improving the well-being of women with a history of hormone receptor-positive (HR^+^) EC (or BC).

## 5. Ovarian Cancer

Ovarian cancer (OC) is a malignant tumor with the worst prognosis of all gynecological cancers. An initial, often promising response to treatment is followed by recurrence in about 80% of women, which then becomes the cause of death for up to 90% of patients [70]. In this context, high levels of HA may be associated with the histologic gradation and pathological type of the tumor [95]. Accumulation of this polysaccharide in ovarian stromal tissue may contribute to enhanced progression in OC, but may also be an independent, prognostic biomarker or potential target for modern cancer therapies. In addition, the hyaluronan-related genes (HAS2, HYAL-1–4, HYALP1, and PH20) are associated with prognosis, cell viability, and spheroid-forming ability in OC [96]. On the other hand, as ICAM-1 has been identified as a potential oncogene that promotes the development of epithelial OC or high-grade serous ovarian carcinoma, and has been found to be associated with poor patients’ survival [97], the HA-mediated reduction in the expression of this specific cell-surface receptor may have anticancer effects.

High stromal HA levels have been associated not only with advanced tumor stage, but also with poor differentiation, serous histological type, or large primary residual tumor in the case of epithelial OC. However, they were not correlated with marked CD44 overexpression [64]. HA levels were higher in tumor cells compared to non-cancerous cells (*p* = 0.001), particularly in stage III tumors (>49-fold) and metastases (>89-fold) [65]. In contrast to elevated HABP1 protein levels possibly being an indicator of lymph node and peritoneal metastasis among women with epithelial OC [68], there was no clear association between the elevated CD44 expression and adverse prognosis for patients with this type of OC [67]. In contrast, an analysis by Sacks and Barbolina [9] indicated a rather ambivalent relationship between the HA-binding CD44 expression and the course of OC. The authors of this study analyzed 23 studies on a diverse number of OC patients (groups ranging from 11 to 483 women) [9]. While some of the studies indicated an association of the CD44 overexpression with a worse prognosis for patients (disease progression and recurrence, metastasis, shorter survival), the results of other studies suggested that the increased expression of this receptor may be associated with improved outcomes of the applied oncological treatment [9]. In addition, several other authors did not observe a correlation between the CD44 receptor status and the clinical course of this type of cancer [9].

A study by Karan Križanac et al. [15] provided slightly different results, which may be closely related to the type (invasiveness) of OC studied. The authors of this study detected the elevated expression of the CD44 protein in 43% of 82 serous OC samples. It was associated with a shorter OS (*p* < 0.001), higher disease stage (stage III/IV), and risk of vascular space invasion [15], and thus, CD44 was identified as a potential and independent prognostic indicator of shorter survival of patients with high-grade OC. According to a recent study by Balduit et al. [98] among high-grade OC patients with stage II/III disease (according to FIGO), as well as on two cancer cell lines (OVCAR-3, SKOV-3), both HA and fibronectin were found in ovarian tissues. HA enhanced the resistance of cancer cells to the applied treatment with cisplatin, while fibronectin, on the other hand, promoted the proliferation and invasion of cancer cells through the induction of ERK and p38 signaling [98].

Ricciardelli et al. [66] conducted a study to elucidate the effect of the CD44 protein on the adverse course of OC. HA levels, CD44 expression, and ABC family protein transporters were studied in 101 OC patients, 22 women with benign tumors, and a group of healthy women; the established OC cell lines (OV-90, OVCAR-3, OVCAR-5, and SKOV-3) were also used in the study [66]. HA levels were determined before treatment, after treatment with carboplatin, and during relapse [66]. It was found that high serum HA levels (>50 μg mL^–1^) were significantly associated with shorter PFS and OS [66]. In addition, serum HA levels were significantly higher in women with OC compared to those in the control group before cancer treatment, while they were not significantly different from HA levels in the group of patients with benign tumors [66]. At the same time, it was found that HA levels in the serum of women with this type of cancer were significantly elevated after at least two cycles of chemotherapy, as well as at the time of the first and the second relapse [66]. A detailed analysis showed that after administration of carboplatin, HA levels increased in 75% of patients; HA, therefore, increased the resistance of tumor cells to the chemotherapy used [66], which is in line with the previously mentioned observations [98]. However, studies on cell lines showed that HA increased the expression of ABC transporters, but only in cancer cells overexpressing the CD44 protein [66]. Similarly to the observations for EC cells [58], HYAL-1 expression was also significantly reduced in OC cells and correlated with HA accumulation [69].

Understanding the key HA–CD44 interactions appears to be crucial to finding ways to overcome tumor cell resistance to chemotherapy and, thus, to developing effective targeted therapies [9,66,99,100]. Lee et al. [101], based on a review of numerous studies, have found that the overexpression of CD44 was evidenced in as many as 90% of OC specimens tested. Since some patients were resistant to classical chemotherapy with paclitaxel, the above authors constructed interesting HA-paclitaxel conjugates with metronomic dosing in an animal model to enhance the penetration of the cytostatic into tumor cells, mediated by the CD44 receptor [101]. The strategy proved effective, in part due to additional anti-angiogenic activity [101]. Nevertheless, further clinical trials targeting the HA-CD44 pathway in OC are needed [9,66,98].

On the other hand, the use of cross-linked hyaluronan gel (CHAG), known for its anti-adhesion properties, inhibited the migration and invasion of OC cells in an animal model mainly through EGFR modulation [102]. Specifically, it has been demonstrated that CHAG blocks the EGF-induced activation of EGFR; inhibits the EGF/EGFR-initiated activation of ERK, Akt, and Rac1; and decreases the EGF-induced expression of PCNA and MMP7 [102]. 

## 6. Conclusions

**Figure 3:**
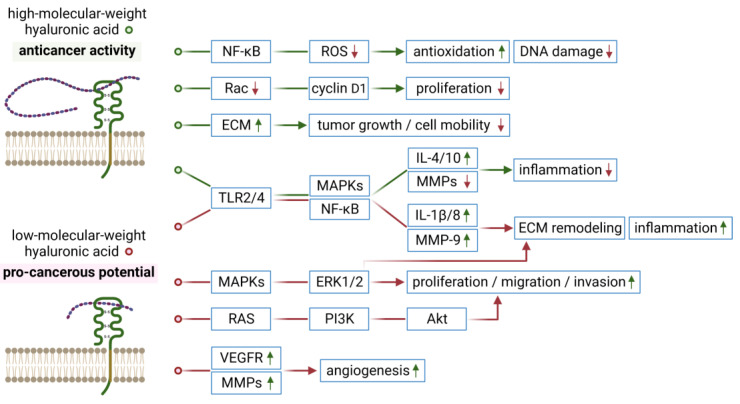
Summary of the anticancer activities of high-molecular-weight hyaluronic acid versus pro-cancerous potential of its degradation products (low-molecular-weight hyaluronic acid) due to triggering the signaling cascades through CD44 receptor. Abbreviations used: ECM, extracellular matrix; ERK1/2, extracellular signal-regulated protein kinases 1 and 2; IL, interleukin; MAPKs, mitogen-activated protein kinases; MMPs, matrix metalloproteinases; NF-κB, nuclear factor κ-light-chain-enhancer of activated B cells; PI3K, phosphoinositide 3-kinase; ROS, reactive oxygen species; TLR2/4, Toll-like receptors 2 and 4; VEGFR, vascular endothelial growth factor receptor. The figure was created with BioRender.com, accessed on 28 November 2022.

Hyaluronic acid (HA) is a unique, non-sulfated glycosaminoglycan, widely distributed in connective, epithelial, and neural tissues. HA plays a role in cell proliferation and migration, but may also contribute to the modulation of growth and functional properties of tumor cells, including their invasion, adhesion, or angiogenesis associated with tumorigenesis at various stages of disease development. It is of note that the effects of HA on tumors depend on the molecular size of this polysaccharide [102]. Specifically, while high-molecular-weight HA (200–2000 kDa) may control normal homeostasis and display anticancer activities [103,104], the products of its degradation (aka low-molecular-weight HA, <200 kDa) show pro-cancerous potential [104]; these opposite effects related to the molecular weight of hyaluronan have been reviewed recently by other authors (Figure 3) [105]. The interactions between HA and its specific cell-surface receptors, mainly CD44, localized on cancer cell membranes may activate several pathways, thereby leading to the promotion of growth of tumor cells and an increase in their metastatic potential.

The results of some studies further indicate that HA levels, as well as the expression of other components of the HA signaling pathway—particularly the CD44 receptor, degradative enzymes (hyaluronidases), and hyaluronate synthases—are promising as very useful biomarkers, not only for prognosis but also for diagnosis or monitoring treatment of cancer patients. In this context, high serum, plasma, or stromal HA levels and overexpression of selected HA family members appear to be associated with poorer prognosis and lower survival in women with selected malignancies. HA is involved in the early development of unfavorable endometrial lesions. At the same time, the accumulation of this polysaccharide correlates with the degree of malignancy in many types of solid tumors, including breast, cervical, endometrial, or ovarian cancer. Under the influence of hyaluronidases, proangiogenic HA breakdown products (oligosaccharides) may be formed, resulting in an increase in the aggressiveness and invasiveness of tumor cells. Increased expression of hyaluronate synthases could be observed, for example, in peripheral areas of tumors derived from highly metastatic breast cancer cell lines.

The HA-CD44 signaling pathway may be a very attractive target in the search for new and promising ways to combat gynecological malignancies, including overcoming the resistance of tumor cells to classical chemotherapy. In addition, the effects of HA-based preparations seem to increase the comfort of life of breast, cervical, or endometrial cancer patients. Additionally, methods of qualitative and quantitative determination of HA levels in biological samples may be interesting diagnostic and/or prognostic tools in the course of selected malignancies among women. Nevertheless, it should be clearly emphasized here that the results obtained to date are quite inconclusive; for this reason, further in-depth studies in this area are needed. The molecular size of HA (i.e., low-molecular-weight HA versus high-molecular-weight HA), the polydispersity of HA products, and the use of HA from different animals or tissues are some of the factors that should be considered when designing new research on this polysaccharide.

## Figures and Tables

**Table 1:** Original studies on the role of HA and its family members in gynecological cancers.

Cancer Type	HA Family Member	Methods	Main Results	Ref.
Breast cancer	HA level	83 women with BC, including 57 with metastatic cancer	High serum levels of HA were found in patients with metastatic disease; lower levels of HA correlated with positive response to classical chemotherapy	[49]
	HA level	48 primary and 212 metastatic BC patients, and 60 healthy women	The median plasma level of HA in patients with metastatic BC was ~two-fold higher than that in primary BC patients and healthy women; plasma HA levels displayed prognostic and treatment monitoring values for women with metastatic variants of disease	[50]
	HA level HASs	50 BC patients and 40 healthy women	Elevated serum levels of HA, together with increased HASs activity, were found in patients prior to chemotherapy compared to the control group; after the first cycle of chemotherapy, HA levels were decreased	[51]
	HABP1	63 BC and non-cancerous tissues	Elevated expression of HABP1 mRNA was found in 41 of 63 primary tumor samples; 5-year survival rates were 29% and 54% in cancer patients with high or low HABP1 mRNA levels, respectively	[52]
	HAS2	Cell lines: MCF-7, MCF-7/DR (drug-resistant cancer cell line)	High expression of HAS2 was responsible for Nrf2 activation; pharmacological inhibition of HAS2 improved the sensitivity of MCF-7/DR cells to the action of doxorubicin; overexpression of HAS2 mediated activation of Nrf2 in drug-resistant cancer cells	[48]
	HAS2	Cell lines: BT-474, BT-549, MCF-7, MDA-MB-231, MDA-MB-453, MDA-MB-468; NOD/SCID mice	HAS2 expression corresponded to the malignant phenotype of BC, and its exogenous expression regulated cell malignant phenotype and invadopodia formation in luminal BC; HAS2-HA signaling was required for the formation of invadopodia in cancer cells; HAS2 promoted both growth and metastatic potential of orthotopically injected luminal BC in the animal model	[53]
	HYAL-1	Cell lines: MCF-7, MDA-MB-231; BALB/C nude mice	HYAL-1 was expressed in BC; knockdown of HYAL-1 expression reduced enzyme activity; inhibited the proliferation, adhesion, invasion as well as potential to angiogenesis in vitro; and led to the inhibition of tumorigenesis of BC in the animal model	[54]
Cervical cancer	CD44s CD44v3	Cell lines: HeLa	CD44s/CD44v3 expressions were inhibited by natural polyphenols; low-molecular-weight HA showed growth-promoting activity in HeLa cells, in contrast to high-molecular-weight HA	[55]
	HABP1	30 CIN, 118 CC specimens, and 10 normal specimens	HABP1 expression was shown to be higher in CC than in high-grade CIN; overexpression of this protein correlated with advanced FIGO stage, poor histologic grade, large tumor size, LVSI, deep stromal infiltration, and lymph node metastasis, and seemed to be an independent factor for disease-free survival	[56]
Endometrial cancer	HA level HAS1–3	Sera obtained from 59 EC patients and 22 postmenopausal healthy women	Serum levels of HA were higher in the EC group than in the corresponding control group; the expression of HAS1 was related to the depth of myometrial invasion, histological grade, and LVSI	[57]
	HA level HAS1–3 HYAL-1–3	39 EC biopsies with different histologic grade (grades I–III)	HA, HASs, and degradative enzymes of HA were identified in EC of all histologic grades; HA was predominantly localized to tumor-associated stroma, particularly to the basal surface of cells	[42]
	HA level HAS1–3 HYAL-1–2	Endometrial tissue specimens collected from 35 patients	The immunoreactivity of HASs was increased in the cancer epithelium; HYAL-2 mRNA was reduced in EC and correlated with HYAL-1; an inverse correlation between HYAL-1 mRNA and the epithelial and stromal HA levels was found	[58]
	HABP1	188 EC, 43 benign endometrial lesions, and 41 normal endometrium specimens	HABP1 was overexpressed in EC and benign endometrial lesions, compared with normal endometrium; cancer patients with high HABP1 expression had a poorer overall and disease-free survival than individuals with low expression of this protein	[59]
	HYAL-1	Endometrial tissue specimens collected from 343 patients	Reduced HYAL-1 expression was associated with the progression of EC towards higher grades and large tumor sizes, lymph node metastasis, and lymphovascular invasion	[60]
	CD44 RHAMM	104 tissue samples of EC	Higher CD44/RHAMM expression correlated with higher depth of myometrial invasion, LVSI, and FIGO stage of disease	[61]
	RHAMM	225 samples of EC and 8 samples of normal endometrium	Increased expression of RHAMM protein was found in EC compared with no or weak expression in normal endometrium; higher RHAMM expression was related to more malignant tumors	[62]
	RHAMM	89 EC and 15 normal endometrium specimens	Increased RHAMM expression was detected in 58% of 89 tumor samples; the positivity rates for RHAMM were 100% and 51% in patients with positive or negative lymph nodes, respectively; RHAMM overexpression correlated with higher histological grade of the tumors and occurrence of lymph node metastases	[63]
Ovarian cancer	HA level	Histological sections of 309 epithelial OC and 45 matched metastatic lesions	High stromal levels of HA corresponded with poor differentiation, serous histological type, advanced stage, and large primary residual tumor, but was not associated with CD44 overexpression on cancer cells; high levels of this polysaccharide were found more frequently in metastatic lesions than in primary tumors	[64]
	HA level HYALs	Ovarian tissue specimens from 78 patients	HA levels increased in cancers, especially in grade III tumors and metastases; HYALs activity slightly decreased from semi-malignant through low-grade to high-grade tumors	[65]
	HA level CD44	Cell lines: OV-90, OVCAR-3/5, SKOV-3; serum from OC patients	HA level was increased following carboplatin treatment and predicted OC outcome; HA treatment increased resistance of OC to chemotherapy; HA regulated expression of ABC transporters	[66]
	CD44	Samples collected from 307 patients with epithelial OC	51% of the tumors had a high proportion of CD44-positive cells; overexpression of CD44 predicted better 5-year overall survival and recurrence-free survival	[67]
	CD44	81 OC tumor sections	CD44 expression was found in 43% of OC samples; the expression of CD44, FIGO stage III and IV, and the presence of vascular invasion was related to a shorter overall survival	[15]
	HABP1	Samples collected from 161 patients with epithelial OC	HABP1 was overexpressed in most metastatic lesions; high expression of HABP1 correlated with peritoneal (95% cases) and lymph node metastases (48% cases) among patients with primary tumors	[68]
	HAS1–3 HYAL-1–2	39 ovarian tissue specimens from 39 patients	HASs expression was not consistently elevated in serous epithelial OC; expression of HYAL-1 was reduced and correlated with the accumulation of HA	[69]

Abbreviations used: BC, breast cancer; CC, cervical cancer; CD44, cluster of differentiation 44; CIN, cervical intra-epithelial neoplasia; EC, endometrial cancer; FIGO, the International Federation of Gynaecology and Obstetrics staging system; HA, hyaluronic acid; HABP1, hyaluronan binding protein 1; HAS(s), hyaluronate synthase(s); HYAL(s), hyaluronidase(s); LVSI, lymphovascular space involvement; OC, ovarian cancer; RHAMM, receptor for hyaluronan-mediated motility.
